# Glutathione-Sensitive Mesoporous Organosilica-Coated Gold Nanorods as Drug Delivery System for Photothermal Therapy-Enhanced Precise Chemotherapy

**DOI:** 10.3389/fchem.2022.842682

**Published:** 2022-02-25

**Authors:** Hui Song, Tingwei Peng, Xue Wang, Beibei Li, Yufang Wang, Dianhai Song, Tianzhao Xu, Xinghui Liu

**Affiliations:** ^1^ Department of Clinical Laboratory, Shanghai Gongli Hospital, The Second Military Medical University, Pudong New Area, Shanghai, China; ^2^ Postgraduate Training Base in Shanghai Gongli Hospital, Ningxia Medical University, Pudong New Area, Shanghai, China

**Keywords:** GSH sensitive, organosilica, GNR, chemotherapy, photothermal therapy

## Abstract

The combination of photothermal therapy (PTT) and chemotherapy can remarkably improve the permeability of the cell membrane and reduce the concentration of chemotherapy agents that not only kill the tumor cells effectively but also have adverse effects on normal tissues. It is of great meaning to construct nanomaterials that could be simultaneously applied for tumor eradication with PTT and chemotherapy. In this work, we developed a novel gold nanorod coated with mesoporous organosilica nanoparticles (oMSN-GNR), which presented as an optimal photothermal contrast agent. Moreover, after doxorubicin loading (oMSN-GNR–DOX), the organosilica shell exhibited biodegradable properties under high glutathione in the tumor microenvironment, resulting in massively releasing doxorubicin to kill tumor cells. More importantly, the hyperthermia effect of GNR cores under near-infrared light provided promising opportunities for localized photothermal ablation *in vivo*. Therefore, the combination of precise chemotherapy and highly effective PTT successfully inhibited tumor growth in liver tumor-bearing mice. This versatile synergistic therapy with local heating and chemotherapeutics precise release opens up the potential clinical application of PTT and chemotherapy therapeutics for malignant tumor eradication.

## 1 Introduction

Photothermal therapy (PTT), unlike traditional photodynamic therapy and radiotherapy, which have the antitumor activity mainly relying on reactive oxygen species generation with intracellular oxygen as its prerequisite, totally exerts its effect *via* increasing the local temperature; undoubtedly, it does not require any tumor microenvironment medium to produce cytotoxicity effect on cancer cells (Dickerson, et al., 2008; Feng, et al., 2015). PTT-related hyperthermia could induce several hazardous effects, such as protein denaturation, cell membrane lysis, and cytoplasmic concentration, thus leading to cell apoptosis (El-Sayed, et al., 2006; Huang, et al., 2006; Kim, et al., 2006). As an ideal tumor treatment modality, PTT can selectively kill tumor cells by hyperthermia based on the heat tolerance variation of normal cells and tumor cells (Hirsch, et al., 2003; Chu and Dupuy 2014; Hwang, et al., 2014). Considering the higher sensitivity of tumor cells toward high temperature, nanoparticle-mediated thermal inhibition with precise accumulation at tumor tissue serves as an optimal treatment means (Hauck, et al., 2008; Park, et al., 2009; Hwang, et al., 2014). However, PTT generally needs access to light sources for direct irradiation, thus precluding the extensive utility for disseminated and metastatic tumors (Nam, et al., 2018). Fortunately, chemotherapy is an alternative treatment for advanced cancer. It remains the therapy cornerstone in the ablation of metastatic cancers or acts as an auxiliary strategy for early tumor surgery (Humber, et al., 2007; Chong, et al., 2014). Therefore, one way of improving therapeutic efficacy has successfully been obtained through a synergistic approach of chemotherapy and hyperthermia combination (Nam, et al., 2013; Zhang, et al., 2014; Chen, et al., 2015; Wang, et al., 2016). Moreover, a study reported that PTT could enhance the antitumor efficacy of chemotherapy, especially in DNA damaging drugs, such as platinum compounds, mitomycin, and doxorubicin (DOX) (Zhang, et al., 2021). However, free antitumor drugs administered directly exhibit unsatisfied therapeutic outcomes and often cause deleterious adverse effects against normal tissues (Cleeland, et al., 2012; Tatonetti, et al., 2012). To overcome these drawbacks, the most attractive strategy is to construct an antitumor drug delivery system, which could realize effectively targeted delivery and precisely controlled drug release, resulting in therapeutic efficacy improvement and adverse effect minimization.

With a localized surface plasmon resonance (LSPR) at the near-infrared region (NIR), gold nanorod (GNR) manifests high photothermal conversion efficiency, making it a promising PTT agent for tumor ablation (Link and El-Sayed 2000; Raschke, et al., 2003; Pérez-Juste, et al., 2005; Hartland 2006; Huang, et al., 2009). However, as a novel theranostic platform, GNR bears two disadvantages: low specific surface area impending the drug loading and the clustering and aggregating properties within cells (Gui and Cui 2012; Hou, et al., 2019). To solve the limitations mentioned earlier, GNR with mesoporous silica coating was developed as a multifunctional theranostic system (Zhang, et al., 2012; Chen, et al., 2015; Ye, et al., 2021). The GNR core functions as the hyperthermia agent and releases heat for inhibiting cancer cell DNA repair (Pan, et al., 2017). The outer shell, mesoporous silica, exhibits the potential capability of high chemotherapy drug payload, thus endowing itself as an optimal drug carrier (Vallet-Regí, et al., 2018; Manzano and Vallet-Regí, 2020). Additionally, biocompatible silica shell protection can guarantee the stability of GNR under complex biological environments. However, drug loading in the pore channels could not be triggered to totally release under tumor microenvironment, substantially limiting its wide application in tumor elimination (Thomas, et al., 2010; Yang, et al., 2012). Above all, a tumor microenvironment-sensitive drug delivery system should be investigated extensively based on the GNR drug delivery system.

**FIGURE 1:**
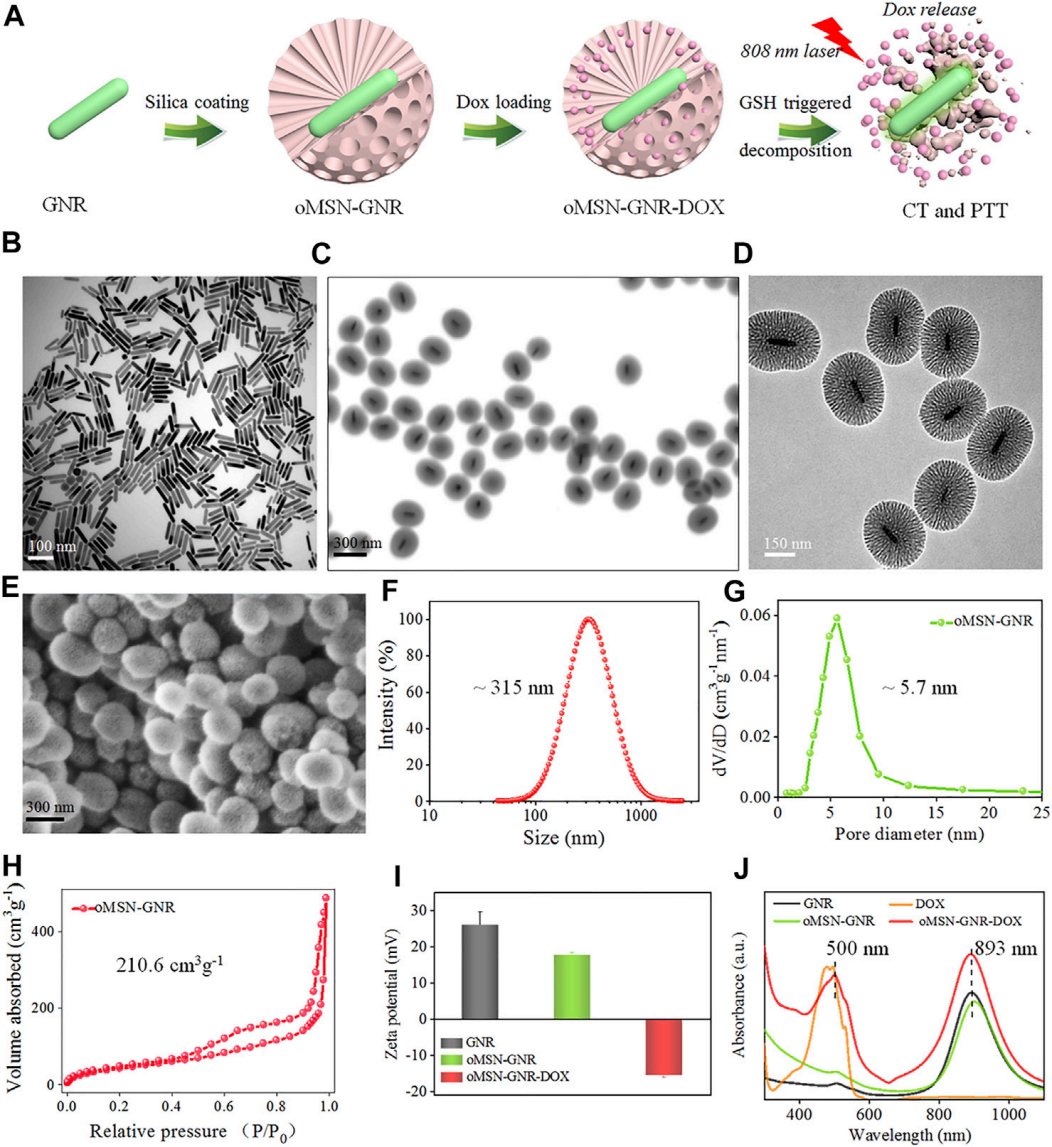
Schematic illustration of oMSN-GNR–DOX fabrication and GSH-triggered DOX release and PTT **(A)**. TEM image of GNR **(B)**, oMSN-GNR **(C),** and magnification TEM image of oMSN-GNR **(D)**. Scanning electronic microscope image **(E)**, size distribution **(F)**, pore size **(G)**, and specific surface area **(H)** of oMSN-GNR. Zeta potential data of GNR, oMSN-GNR, and oMSN-GNR–DOX **(I)**. Ultraviolet–visible spectrum of DOX, GNR, oMSN-GNR, and oMSN-GNR–DOX **(J)**.

In this work, GNR with mesoporous organosilica core–shell nanostructure (oMSN-GNR) was fabricated for the first time. Owing to the high surface area and mesopore channels of mesoporous silica, DOX could be efficiently encapsulated (oMSN-GNR–DOX). Owing to the tetrasulfide bond in the framework, the silica shell presents biodegradation capability under high glutathione (GSH) in tumor cells, leading to a sharp release of DOX for DNA damage. More importantly, high temperatures with ∼75°C have been obtained for tumor cell eradication (Figure 1A). In this method, the oMSN-GNR–DOX is capable of realizing the effective antitumor drug accumulation at the tumor site *via* the conventional enhanced permeability and retention effect. Then, under the synergistic effect of PTT and chemotherapy, liver cancer had been totally eliminated. This multimode therapy platform holds great potential application in solid tumor eradication.

## 2 Materials and Methods

### 2.1 Synthesis of Gold Nanorod Coated With Mesoporous Organosilica Nanoparticle-Doxorubicin

#### 2.1.1 Gold Nanorod Fabrication

GNR with an LSPR peak at ∼900 nm was synthesized *via* a general seed-mediated strategy in an aqueous system. Briefly, 0.6-ml NaBH_4_ (10 mM) was prepared on the ice-cold bath. Then, it was injected into an aqueous solution (10 ml) containing cetyltrimethylammonium bromide (CTAB; 0.1 mol/L) and HAuCl_4_ (0.25 mmol/L) under vigorously stirred. As seeds, gold nanospheres were acquired after a 2-min reaction, and the solution discussed was always kept open for 0.5 h at room temperature to facilitate NaBH_4_ hydrolysis. Then, GNR growth solution was subsequently prepared by adding 0.2-ml HAuCl_4_ (25 mmol/L) to 10-ml CTAB (0.1 mol/L). Next, 40-μl AgNO_3_ (16 mmol/L) and 90-μl L-ascorbic acid (80 mmol/L) were separately mixed into the system. The GNR growth solution changed into colorless after continuous handshaking, and 12-μl previously synthesized gold seeds were rapidly injected into the growth solution. The solution was kept undisturbed for 18 h at 37°C to contribute to the GNR growth. The as-prepared GNR was then washed by centrifugation more than two times (7,200 rpm, 10 min), and the GNR sample was re-dispersed in water.

#### 2.1.2 Gold Nanorod Coated With Mesoporous Organosilica Nanoparticle Fabrication

The tetrasulfide bond co-doped mesoporous organosilica shell coating on GNR was synthesized by a common bi-phase (oil-water) stratification system. According to the reported strategy, the doped mesoporous organosilica nanospheres were fabricated with some modification. Briefly, 1.0-g CTAB and 100-mg GNR were firstly re-dispersed in a 60-ml deionized water solution. After, CTAB completely dissolved under 60°C continuous stirrings. Latterly, 750-μl 25% triethylamine was immediately mixed to reaction system mentioned earlier at three necks round-bottom flask with vigorously stirring for 1 h in 60°C oil bath. Simultaneously, 20 ml of tetraethyl orthosilicate (12%) and bis(triethoxysilyl) (8%) in cyclohexane mixture was carefully added drop by drop on the surface of the aqueous solution discussed earlier. With 24 h gently stirring, the core–shell oMSN-GNR sample was obtained after careful separation of the upper layer cyclohexane *via* pipette. The precipitate of the as-synthesized sample was acquired after centrifuge treatment (10,000 rpm, 0.5 h). The final oMSN-GNR must be washed by water or ethanol more than three times. Latterly, the product discussed earlier was added into 0.6% NH_4_NO_3_/ethanol solution at 100-ml one-neck round bottle flask *via* strong ultrasonic administration. Therefore, residual CTAB was finally removed after 12-h treatment in a 60°C oil bath. The core–shell nanocomposites were obtained after being alternately washed by water and ethanol more than three times. The obtained oMSN-GNR was re-dispersed in water.

#### 2.1.3 Doxorubicin Loading in Gold Nanorod Coated With Mesoporous Organosilica Nanoparticles

DOX was firstly dissolved in 2.0-ml dimethyl sulfoxide. oMSN-GNR nanoparticles (∼5.0 mg) were added to the dimethyl sulfoxide solution, and then, it was stirred for 48 h at room temperature. The DOX molecules can be adsorbed and stacked in mesoporous channels of the organosilica shells. The obtained DOX-loaded oMSN-GNR nanoparticles were collected *via* centrifugation. The amount of the absorbed drugs was determined from the supernatant liquid percentage in the initial amounts of DOX after being measured by the ultraviolet–visible absorbance and then quantified by a standard curve of DOX concentration *vs.* absorption. The indocyanine green (ICG) loading procedure was similar to that of DOX loading.

### 2.2 *In Vivo* Synergic Therapy

#### 2.2.1 *In Vivo* Tumor Eradication Efficiency

Liver cancer-bearing BALB/c nude mice were randomly divided into four groups and subjected twice to intravenous injection for 15 days of tumor ablation. Accordingly, the different treatments (*n* = 4 per group): (1) phosphate-buffered saline (PBS), (2) DOX, (3) oMSN-GNR–DOX, and (4) oMSN-GNR–DOX + laser. The laser irradiation was performed 24 h after tail vein injection (808 nm laser, 1 W/cm^2^, 5 min). All dose of the GNR-based platform was set as 8 mg kg^−1^, and the DOX group received the same concentration of DOX as the oMSN-GNR–DOX group. All groups had taken digital photos every 3 days. The tumor size was carefully measured *via* a digital caliper. Tumor volume was calculated by a common formula (width × width × length × 0.5). Meanwhile, body weight was detected every 3 days.

#### 2.2 2 Histopathological Evaluation

Tumors of PBS, DOX, oMSN-GNR–DOX, and oMSN-GNR–DOX + laser treatment for 5 days were dissected and then carefully cut into ∼10-μm-thick tissue slices. Hematoxylin and eosin (H&E) analysis and terminal deoxynucleotidyl transferase deoxyuridine 5-triphosphate nick end labeling (TUNEL) staining were subsequently performed. Meanwhile, the major organs (heart, liver, spleen, lung, and kidney) after PBS, DOX, oMSN-GNR–DOX, and oMSN-GNR–DOX + laser treatment for 15 days were also evaluated by H&E staining.

## 3 Results and Discussion

### 3.1 Gold Nanorod Coated With Mesoporous Organosilica Nanoparticle Fabrication and Characterization

GNR was obtained *via* a common seed-mediated growth strategy according to the previous reports (Shen, et al., 2014). After regulating the concentration of seeds, silver ions for size, and pH value for LSPR, ∼100-nm GNR with a peak at ∼900 nm was synthesized. Transmission electron microscopy (TEM) image results showed the GNR nanocrystals with uniform size and excellent dispersion (Figure 1B). According to a reported method of bio-phase (water/cyclohexane) system, organosilica mesoporous silica was coated on GNR (Yang, et al., 2013). During the silica-coating procedure, CTAB formed as the surfactant around the GNR and served as the single micelle template for the formation of the mesoporous organosilica silica shell. The ratio of conventional silica precursor (tetraethyl orthosilicate) and organosilica precursor [bis-(triethoxysilylpropyl) tetrasulfide)] was set as 4:1. After 48-h reaction at 60°C, oMSN-GNR with the size of ∼300 nm was successfully fabricated, as shown in the TEM image of Figure 1C, the synthesized core–shell nanosphere could be clearly observed, and the amorphous silica shell was evaluated to present a homogeneous thickness with ∼100 nm. Disordered mesopores of ∼5 nm in diameter could be distinctly estimated from TEM in Figure 1D and scanning electronic microscope image in Figure 1E, offering an ideal opportunity for oMSN-GNR to be performed as general drug delivery carriers. Moreover, the dynamic laser scanning result exhibited that the average size of oMSN-GNR was estimated as ∼315 nm (Figure 1F) with pore size evaluated as ∼5.7 nm after calculating by nitrogen adsorption branch of the isotherm *via* the density functional theory approach (Figure 1G). Importantly, insignificant variation of size distribution was found after different biological buffer treatments (such as PBS, 10% blood, fetal bovine serum, and cell culture medium) for different hours, suggesting the superior stability of GSH-sensitive mesoporous nanoshell nanoparticles (Supplementary Figure S1). Meanwhile, the specific surface area of as-synthesized oMSN-GNR was analyzed as ∼210.6 cm^3^ g^−1^ through the conventional Brunauer–Emmett–Teller method (Figure 1H). Both mesoporous channels and appreciable specific surface area facilitated the therapeutic agents' loading and delivery. Subsequently, DOX was then loaded into the mesoporous channels of the organosilica shell at pH 7.4 *via* electrostatic interactions, and the oMSN-GNR–DOX complex was formed after a 12-h reaction. Owing to –Si-OH on the surface of silica, the zeta potential decreased after organosilica coating. Interestingly, zeta potential reversed into the negative side after drug encapsulation, which is mainly ascribed to the negative charge of DOX (Figure 1I). The results demonstrated that massive chemotherapy agents were successfully loaded, and the negative charge could facilitate cellular endocytosis. Simultaneously, ∼10-nm LSPR red-shift was observed after silica coating, and a further small red-shift (∼5 nm) absorption of the characteristic peak of DOX accompanying slight band damping was also observed in oMSN-GNR–DOX, further proving that DOX was successfully encapsulated in the mesoporous shell (Figure 1J).

### 3.2 Glutathione-Sensitive Degradation and *In Vitro* Photothermal Efficiency

**FIGURE 2:**
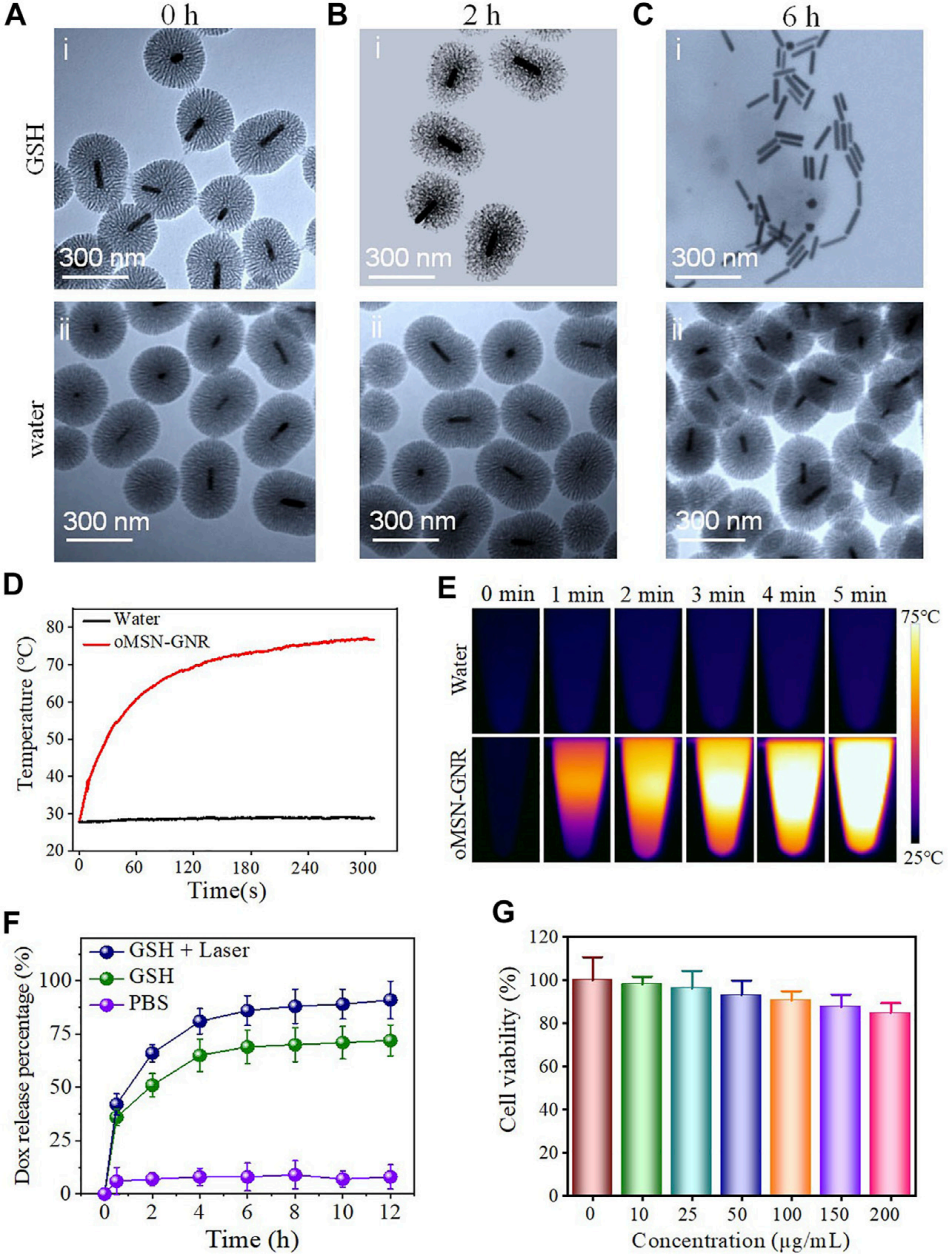
TEM image of oMSN-GNR incubated with 10-mM GSH **(Ai–Ci)** or water **(Aii–Cii)** for 0, 2, and 6 h after NIR laser illumination for 5 min. Temperature rise curves of water and aqueous oMSN-GNR nanocomposites upon exposure of NIR laser for different minutes **(D)**. Photothermal images of water and aqueous oMSN-GNR nanocomposites upon exposure of NIR laser for different minutes **(E)**. Cumulative DOX release behavior of oMSN-GNR–DOX in water, GSH, and GSH + laser group **(F)**. Cell viability of Huh7 cells after co-incubation with oMSN-GNR at different concentrations for 24 h **(G)**.

A multifunctional drug carry system can directionally deliver drug molecules to tumor sites and should precisely release them under specific conditions of different stimuli in tumor cells (Yang et al., 2018; You et al., 2018). Among the different tumor-specific stimuli, imbalance of redox status in tumor cells is an effective initiator for the cytoplasm burst release of drugs. Due to this, redox responsive drug release systems have been developed extensively. Among the redox-sensitive bonds, the tetra-sulfide bond is the most commonly involved in redox disrupted linkages (Reddy, et al., 2005; Koo, et al., 2008). It can be very stable in an extracellular microenvironment with ∼2-μM GSH, but it is capable of being rapidly broken *via* exchange reactions of thiol-disulfide in the cytoplasm with ∼10-mM GSH (Cheng, et al., 2011; Bahadur, et al., 2014). Accordingly, organosilica shell reaction with GSH buffer was firstly investigated. As shown in Figures 2A–C, after 2-h GSH incubation and 808-nm laser irritation, silica shell became soft and loose and completely degraded with only residual GNR remaining after incubated with GSH buffer for 6 h, demonstrating a time-dependent biodegradable behavior under tumor redox microenvironment (Figures 2Ai–Ci). In contrast, in the control group of water incubation, the morphology of the oMSN has negligible transformation even for 6-h coculture (Figures 2Aii–Cii). In addition, the biodegradable rate of oMSN-GNR is faster than traditional mesoporous silica with total decomposition for nearly 5 days, possibly due to heat from GNR that facilitates the redox-sensitive reaction. Meanwhile, considering the GNR exhibits absorption coefficients, the photothermal effect for GNR-based nanocomposites was subsequently estimated in an aqueous solution after 808-nm diode NIR laser illumination. As we can clearly see in Figures 2D,E, after NIR laser irradiation for 2 min, the temperature of oMSN-GNR nanoparticles with concentration at 200 μg/ml drastically increased to ∼70°C and reached the maximum temperature at ∼75°C for 5-min exposure. In contrast, the pure water group maintained the room temperature, even after 5-min irradiation. Taking together, all these hyperthermia evidence proved that the core–shell nanoparticles had a surprisingly optimal photothermal effect for tumor ablation. DOX release behavior was then investigated by ultraviolet–visible spectrum under different buffers at 37°C. We found that in the absence of NIR laser irradiation and GSH treatment, the cumulative DOX release is ignorable even after 12-h incubation, suggesting that electrostatic interaction and π−π stacking force could induce little drug leakage in our system. Interestingly, in comparison with the GSH treatment group with ∼60.12% drug release in 4 h, a faster ∼89.1% DOX release was obtained after irradiation with a 300-mM NIR laser (with 808-nm wavelength) (Figure 2F). Except for GSH triggered silica framework decomposition, under the NIR laser irradiation, the DOX release behavior became the fastest for pure GSH and water treatment. This is mainly attributed to two aspects, laser-concerted hyperthermia could dissociate the strong connection between organosilica and DOX, ^[28]^ and furthermore, the corresponding heat was capable of boosting the reaction between GSH and the co-doped tetra-sulfide bond. Finally, the biocompatibility of GNR-based nanocarriers was studied against liver tumor cell lines. The viability of tumor cells maintained up to 90% even cocultured nanocarrier's concentration reached as high as 200 μg/ml for 24 h, proving the superior biocompatibility and its further application for *in vivo* tumor eradication (Figure 2G).

### 3.3 Cellular Internalization and *In Vivo* Photothermal Therapy Efficiency

**FIGURE 3:**
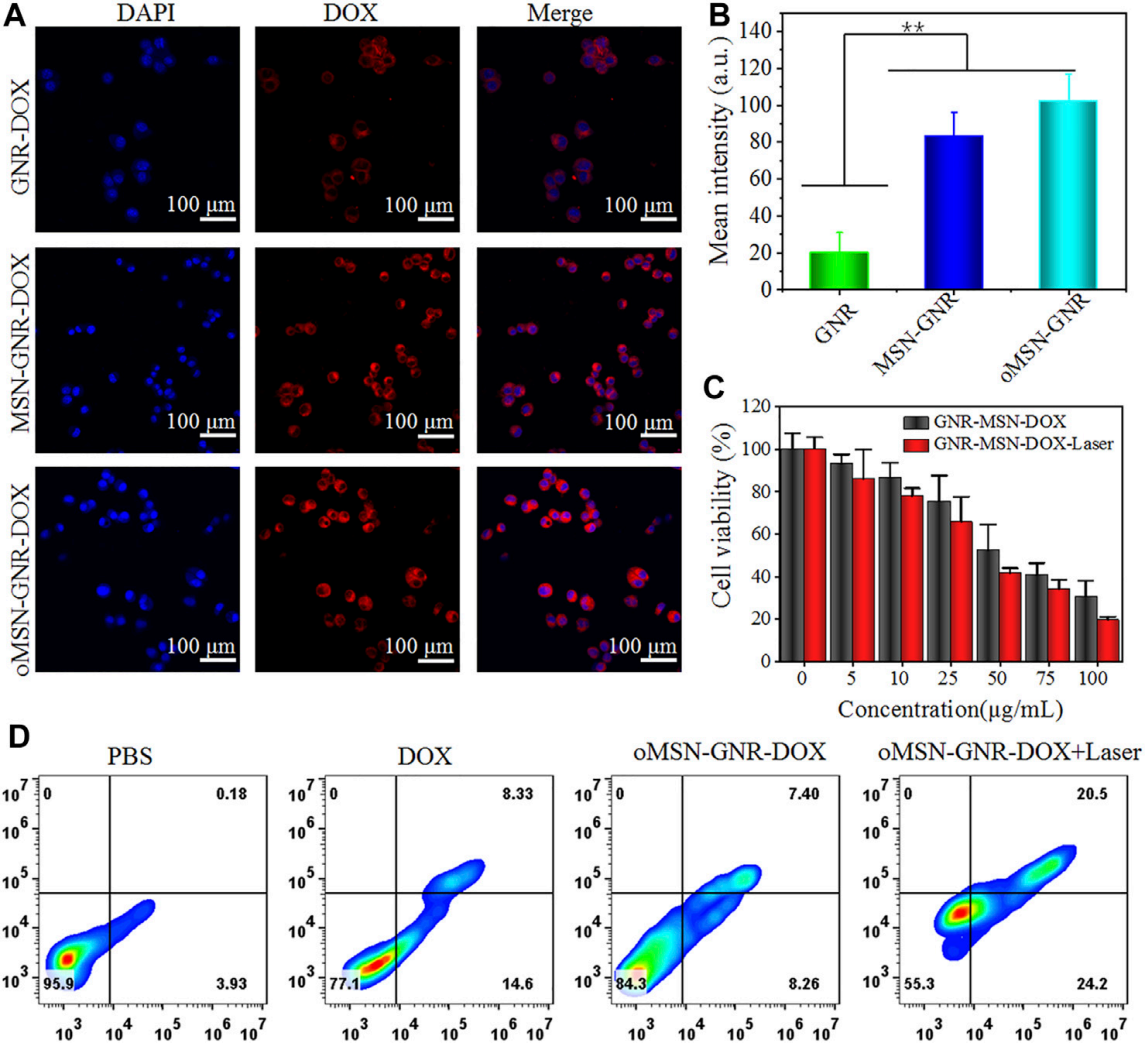
Confocal laser scanning images of huh7 cell internalization after cocultured with GNR-DOX, oMSN-GNR–DOX, and MSN-GNR-DOX for 8 h **(A)**. Quantitative analysis of corresponding mean fluorescence intensity in A **(B)**. Cell viability of huh7 cells after being treated with various concentrations of oMSN-GNR–DOX and oMSN-GNR–DOX + laser for 24 h **(C)**. Cell apoptosis/necrosis after different treatments of PBS, free DOX, oMSN-GNR–DOX, and oMSN-GNR–DOX + laser for 24 h **(D)**. Mean ± standard deviation (*n* = 5), **p < 0.01 *vs.* GNR-DOX, Student t-test.

Inspired by the ideal biocompatibility, we explored the cellular internalization of our GNR-based nanocomposite toward the huh7 cell line. Firstly, DOX was absorbed on the GNR surface through charge adsorption (GNR-DOX), and common mesoporous silica-coated GNR was prepared for DOX loading (MSN-GNR–DOX). During the endocytosis experiment, the GNR concentration of GNR-DOX, oMSN-GNR–DOX, and MSN-GNR–DOX was equivalent. The cytoplasm fluorescence of DOX in GNR-DOX was remarkably lower than MSN-GNR–DOX and oMSN-GNR–DOX, resulting from unsatisfactory drug loading efficiency of free GNR (Figures 3A,B). Moreover, analogous DOX fluorescence can be observed in oMSN-GNR–DOX and common MSN-coated GNR-based DOX carriers, suggesting that tetra-sulfide bond doping in the silica scaffold had a negligible effect on drug loading capacity. Latterly, the cytotoxicity of different concentrations of oMSN-GNR–DOX toward huh7 cells with/without 808-nm laser irradiation was carefully studied. As shown in Figure 3C, concentration-dependent cell viability was detected in both groups. However, after 5 min of 808-nm NIR laser exposure, fewer tumor cells were alive, especially with high concentration treatment in comparison with no laser irradiation. This result could be ascribed to the chemotherapy enhancement *via* hyperthermia, demonstrating the advantage of the synergistic effect of PTT and chemotherapy in our drug delivery platform. Simultaneously, after being treated with PBS, free DOX, oMSN-GNR–DOX, and oMSN-GNR–DOX + laser, cells were then stained by apoptosis assay, annexin V–fluorescein isothiocyanate/propidium iodide before flow cytometry analyzing analysis. We found that in comparison with other groups, the oMSN-GNR–DOX + laser treatment group exhibited the highest percentage of apoptotic/necrotic tumor cells. In contrast, the percentage of apoptotic/necrotic tumor cells in the oMSN-GNR–DOX group was only one-third of that in the oMSN-GNR–DOX + laser treatment group (Figure 3D, Supplementary Figure S2). This flow cytometry results were consistent with cell killing assays, further demonstrating the optimal tumor cell ablation capability of a combination of PTT and chemotherapy.

### 3.4 Tumor Accumulation Capability of Gold Nanorod Coated With Mesoporous Organosilica Nanoparticles

**FIGURE 4:**
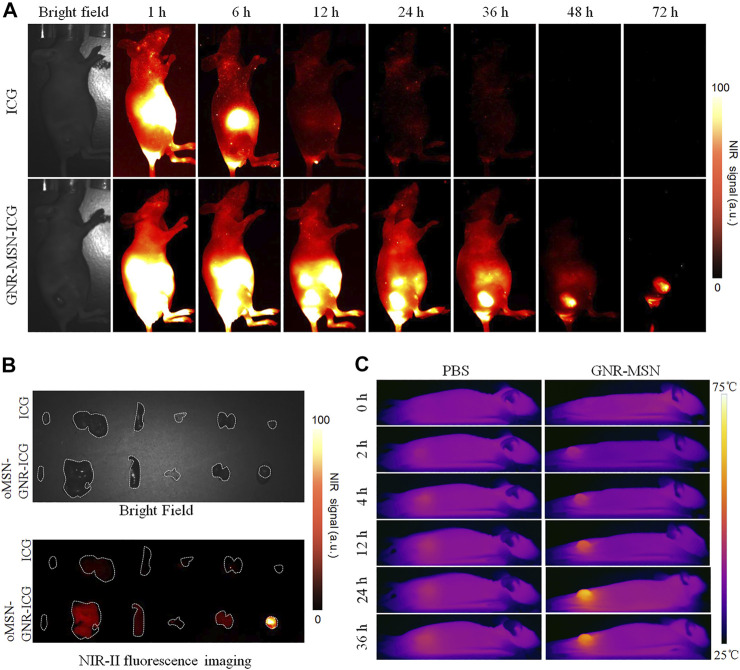
NIR-II fluorescence bioimages of huh7-bearing nude mice after intravenous injection of free ICG or oMSN-GNR–ICG for various periods **(A)**. *Ex vivo* bright-field image (upper), NIR-II fluorescent image (lower) of major organ and tumors after tail injection of free ICG or oMSN-GNR–ICG for 24 h **(B)**. Photothermal images of huh7-bearing mice after tail vein injection of PBS or oMSN-GNR–ICG for various periods. Photothermal photos were obtained after 5 min of 808-nm laser irradiation at each time point **(C)**.

Encouraged by the *in vitro* results discussed earlier, before *in vivo* tumor ablation investigation, we firstly estimated tumor passive targeting capability of our GNR-based nanocarriers *via* enhanced permeability and retention effect in huh7 tumor-bearing mice. To realize high penetration depth and low autofluorescence imaging modality, clinically approved ICG was encapsulated into the organosilica shells to act as NIR-II (the second NIR, 1,000–1,700 nm) fluorescent contrast agents (oMSN-GNR–ICG). After tail vein injection of oMSN-GNR–ICG, NIR-II signals in tumor site could be observed after 6-h post-injection. Surprisingly, tumor tissue had the strongest fluorescence intensity and distinct profile within 24-h post-injection. Meanwhile, tumor signals continuously weakened from this time point and still existed after intravenous injection at 72 h, suggesting that laser illumination for PTT should be carried out at this massive tumor accumulation time (Figure 4A). Moreover, the resected major organs and tumors also displayed that maximum tumor accumulation could be detected at 24-h post-injection (Supplementary Figure S3). In contrast, the free ICG group presented no NIR-II signals in the tumor site, demonstrating that oMSN-GNR–ICG was capable of predominantly improving the tumor-targeting ability and possessed longer tumor retention behavior (Figure 4A). Based on this result, we then collected the major organs and tumors after 36-h treatment of free ICG and oMSN-GNR–ICG to perform *ex vivo* NIR-II fluorescence imaging. Undoubtedly, tumor signals in GNR-based nanoparticle groups exhibited stronger signals in comparison with the free ICG group, which was consistent with *in vivo* NIR-II imaging results (Figure 4B). Finally, owing to the facile accessibility and simple manipulation of the photothermal camera and NIR laser device, photothermal imaging was performed on the mice after being intravenously injected with oMSN-GNR–ICG. The liver tumor exhibited a slight temperature increase in the PBS group (6.2°C) during the whole imaging durations. Contrarily, in the oMSN-GNR–ICG group, the tumor tissue exhibited significantly higher temperature at each time point in comparison with the PBS group. The highest value at 24-h post-injection was detected as 75.2°C, further demonstrating superior passive targeting behavior of this GNR core–shell nanoparticle (Figure 4C).

### 3.5 *In Vivo* Synergistic Therapy Evaluation

**FIGURE 5:**
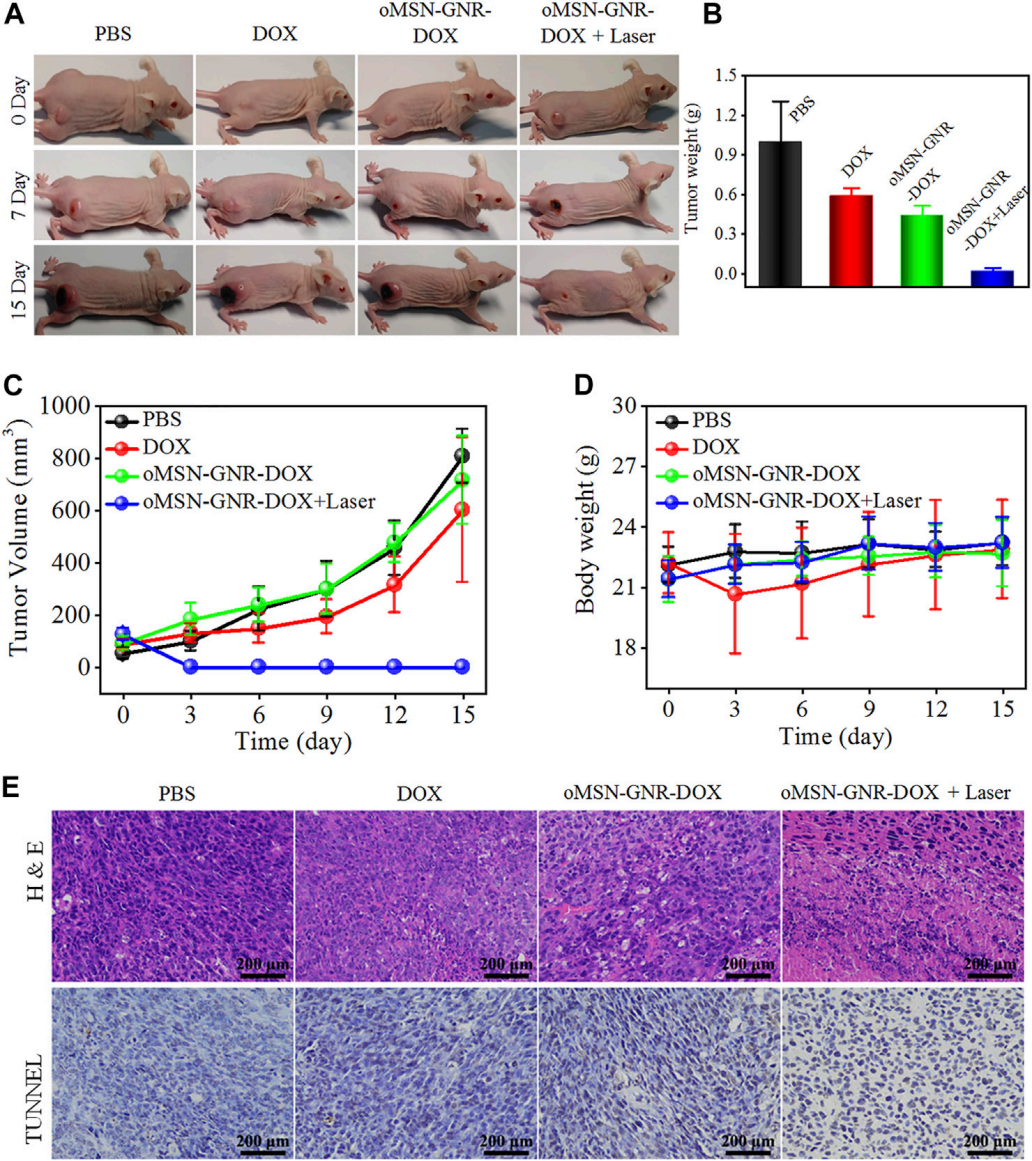
Typical digital images of huh7 tumor-bearing nude mice after treatment of PBS, DOX, oMSN-GNR–DOX, or oMSN-GNR–DOX + laser for 15 days **(A)**. Tumor weight **(B)**, tumor volume curves **(C)**, bodyweight fluctuation curves **(D)**, hematoxylin and eosin (upper), and terminal deoxynucleotidyl transferase deoxyuridine 5-triphosphate nick end labeling (lower) staining images **(E)** of huh7 tumor-bearing nude mice after treatment of PBS, DOX, oMSN-GNR–DOX, or oMSN-GNR–DOX + laser for 5 days.

After determining the time point of the highest tumor targeting ability, the antitumor efficacy of oMSN-GNR–DOX was tested. Liver tumor-bearing nude mice were randomized into four groups (*n* = 4) which were undergone with the following administrations: PBS, DOX, MSN-GNR-DOX, and MSN-GNR-DOX + laser. The important data to estimate tumor therapeutic effects, including tumor weight, tumor volume, and body weight, were recorded every 3 days. The digital images presented that tumor size dramatically increased in the PBS group over the whole inhibition time. Compared with the PBS group, some inhibition effects had displayed in the free DOX and oMSN-GNR–DOX groups. Excitingly, the tumor almost disappeared in the oMSN-GNR–DOX + laser group, which was much more effective than the single chemotherapy group (Figure 5A, Supplementary Figure S4). Tumor weight and tumor volume curve showed that oMSN-GNR–DOX + laser treatment exhibited significantly higher tumor elimination capability (Figures 5B,C), showcasing excellent therapeutic efficiency when combining GSH-sensitive chemotherapy with PTT. Furthermore, tumor slices were prepared for H&E staining. As displayed in Figure 5E, the oMSN-GNR–DOX + laser group obviously exhibited massive tumor cell interspace and lots of abnormal cellular morphology, which was consistent with the TUNEL image, indicating that tumor cells suffered from synergistic cytotoxicity of hyperthermia and DNA damage in the oMSN-GNR–DOX + laser group (Figure 5F). Furthermore, in comparison with the oMSN-GNR–DOX + laser groups, partial antitumor effects could be observed in oMSN-GNR + laser, whereas no significant tumor inhibition performance was detected in the pure laser group, hinting at the ideal antitumor effect of synergistic therapy in oMSN-GNR–DOX + laser treatment (Supplementary Figure S5). Simultaneously, the 808-nm laser has no adverse effect on the skin after 5-min irradiation under 1 W/cm^2^, hinting at the biosafety of this NIR laser for PTT (Supplementary Figure S6). No surrounding tissue damage was observed after PTT, indicating the little adverse effect of PTT against normal tissues (Supplementary Figure S7). More importantly, the bodyweight of all mice in four groups had little fluctuation during 15 days of treatment (Figure 5D); meanwhile, the main organs of each group were finally excised for pathological analysis. Compared with the PBS group, unnoticeable histomorphology changes were found in the other three groups, proving that our GNR-based nanocarriers had ignorable adverse effects, thus acquiring potential clinical application value in the future (Supplementary Figure S8). Finally, all corresponding blood biochemistry (Supplementary Figures S9A–E) and blood routine factors (Supplementary Figures S9F–J) in oMSN-GNR–DOX + laser after 15-day treatment presented negligible fluctuations in comparison with the PBS group, demonstrating the low systematic biotoxicity of oMSN-GNR–DOX for synergistic therapy.

## 4 Conclusion

In this work, we developed a multifunctional therapeutic agent for combination therapy in malignant tumors. This novel nanocarrier system was constructed by a GNR core and mesoporous organosilica shell for the first time. The inner GNR has LSPR at NIR, which could be used as an optimal hyperthermia agent. The outer layer silica exhibits GSH-sensitive biodegradation performance for precise delivery and release of chemotherapeutic drugs. Additionally, the heat could also facilitate DOX bursting release. Tumor cells can be effectively killed under the synergistic effect of PTT and chemotherapy. More importantly, oMSN-GNR–DOX exhibited significant tumor accumulation and predominant tumor inhibition under 808-nm laser irradiation *in vivo* with negligible adverse effects. In summary, as an ideal therapeutic agent, GNR with mesoporous organosilica coating has potential for PTT and chemotherapy combined therapy in clinical.

## Data Availability

The original contributions presented in the study are included in the article/Supplementary Material, further inquiries can be directed to the corresponding authors.
