# Supplementary material for: Glutathione-Sensitive Mesoporous Organosilica-Coated Gold Nanorods as Drug Delivery System for Photothermal Therapy-Enhanced Precise Chemotherapy
Source: Front Chem. 2022 Feb 25;10:842682. doi: 10.3389/fchem.2022.842682 (PMC8914165; doi:10.3389/fchem.2022.842682)
Supplement: Supplementary file 1 [file DataSheet1.docx]

Supplementary Material

# 1.Supplementary Experimental Section

**1.1 Materials and characterization.**

***1.1.1Materials.*** Hexadecyltrimethylammonium bromide (CTAB) and hydrogen tetrachloroaurate(III) trihydrate (HAuCl_4_·3H_2_O), sodium borohydride (NaBH_4_), silver nitrate (AgNO_3_), L-ascorbic acid were purchased from Sigma-Aldrich. NaOH, cyclohexane and NH_4_NO_3_ were obtained from Shanghai Chemical Co., Ltd. Ammonia aqueous solution (28 wt %), tetraethyl orthosilicate (TEOS), (3-Aminopropyl) triethoxysilane (APTES), bis[3-(triethoxysily)propyl] tetrasulfide (BTES), triethanolamine (TEA), decahydronaphthalene (98 %) were purchased from Aladdin Industrial Inc. All chemicals were used as received without further purification. Doxorubicin hydrochloride and tris(hydroxymethyl)aminomethane (Tris) were purchased from Aladdin Industrial Inc. Deionized (DI) water was used in all experiments.

***1.1.2Characterization.*** Transmission electron microscopy (TEM) measurements were carried out on a JEM 2100F microscope (Japan) operated at 200 kV. LEO1530VP SEM (Germany), a Bruker Multimode 8, high-resolution transmission electron microscope operated at 200 kV. SEM measurement was analyzed using Nanoscope V multimode atomic force microscope. The samples were first dispersed in ethanol and then collected by using copper grids covered with carbon films for measurements. UV–vis–NIR absorption spectra were measured on Shimadz spectrophotometer (UV-3150) (Japan) with a wavelength range of 300 –1200 nm, unless otherwise specified, all spectra were collected under identical experimental conditions.

**1.2 *In vitro* cellular targeting and cell killing of oMSN-GNR-DOX**

***1.2.1. Cell viability.***

All experiments were carried in 96-well plates. Cytotoxicity of oMSN-GNR was tested *via* CCK-8 assay. Briefly, the primary huh7 tumor cells isolated through enzymatic digestion were seeded into a plate at 5×10^3^/well in 100 μL of 1640 (10% FBS, 100 units/mL of penicillin and 100 μg/mL of streptomycin), and incubated for 24 h. Then, oMSN-GNR with various concentrations was added followed by 24 h incubation. Lately, 10 μL CCK-8 was added to the cells, and after 2 h incubation, the absorbance of each well at a wavelength of 450 nm was measured using a microplate reader. Data were presented as mean ± SD (n = 3).

***1.2.2. In vitro PTT efficiency evaluation.***

Cell killing efficacy was evaluated after NIR laser irradiation. Briefly, the primary huh7 tumor cells were isolated by enzymatic digestion and then seeded into 96-well plates (5×10^3^/well) in 100 μL of fresh 1640 medium (10% FBS, 100 units/mL of penicillin, 100 μg/mL of streptomycin) for 24 h incubation. Then, oMSN-GNR-DOX with various concentrations were added and incubated for another 24 h. Then cells were irradiated by 808 nm laser for 5 min (1.0 W/cm^3^), and live-cell percentage was detected by CCK-8 assay.

***1.2.3. CLSM images of cellular uptake.***

The cellular uptake of oMSN-GNR-DOX was investigated and imaged by confocal laser scanning microscopy (CLSM). Briefly, huh7 liver tumor cells were firstly seeded into 6-well plates at a concentration of 1×10^5^ per well with 1 mL 1640 fresh medium (10% FBS, 100 units/mL of penicillin, 100 μg/mL of streptomycin) for 24 h incubation. After the treatment with GNR-DOX, MSN-GNR-DOX, oMSN-GNR-DOX at the same concentration of GNR (200 μg/mL), the huh7 liver cancer cells were incubated for 8 hours. Then, all the cell samples were washed by PBS 3 times, and 1 μg /mL DAPI was subsequently used to stain cellular nuclei for 0.5 h before CLSM observation.

***1.2.4 Live/Dead cell staining and cell apoptosis analysis***

Annexin V-FITC/PI apoptosis experiment was also carried out to evaluate the *in vitro* anti-tumor capability and mechanism. Huh7 tumor cells were seeded into 6-well plate at a density of 1×10^5^per well with 1 mL of 1640 fresh media (10% FBS, 100 units/mL of penicillin, 100 μg/mL of streptomycin) for 24 h co-culture, then the tumor cells were administrated by incubation of various formulations: PBS, DOX, oMSN-GNR-DOX and oMSN-GNR-DOX + laser. For the cell apoptosis/necrosis evaluation, Annexin V-FITC/PI apoptosis assay was used to co-culture with above group, respectively. The percentage of cellular apoptosis was further estimated by a flow cytometer.

**2. Supplementary Figures Section**


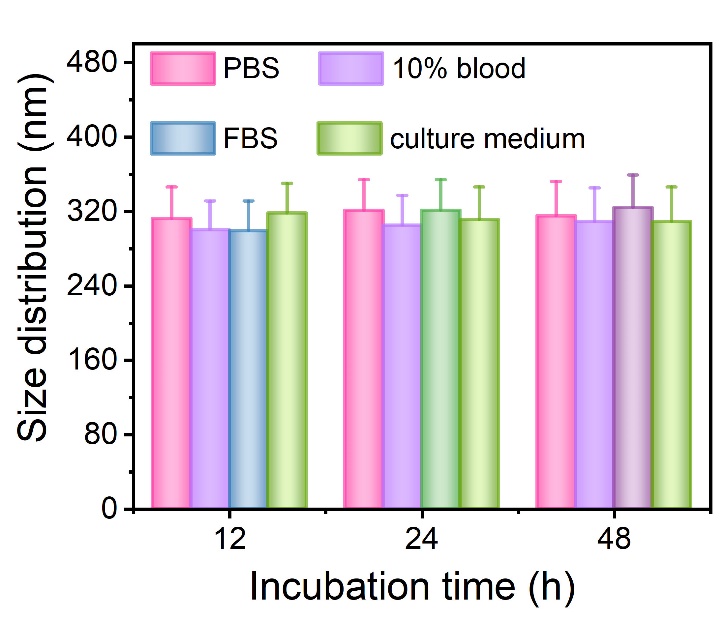


**Figure S1.** Size distribution of oMSN-GNR after incubation with PBS, 10% blood, FBS and culture medium for 12, 24 and 48 h.


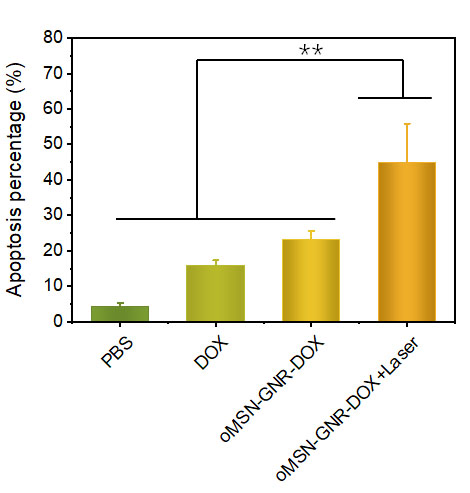


**Figure S2.** Statistical results of flow cytometry after of PBS, DOX, oMSN-GNR-DOX and oMSN-GNR-DOX + laser treatments. Mean ± SD (n= 5), **P< 0.01 vs. oMSN-GNR-DOX + Laser, t-student test.


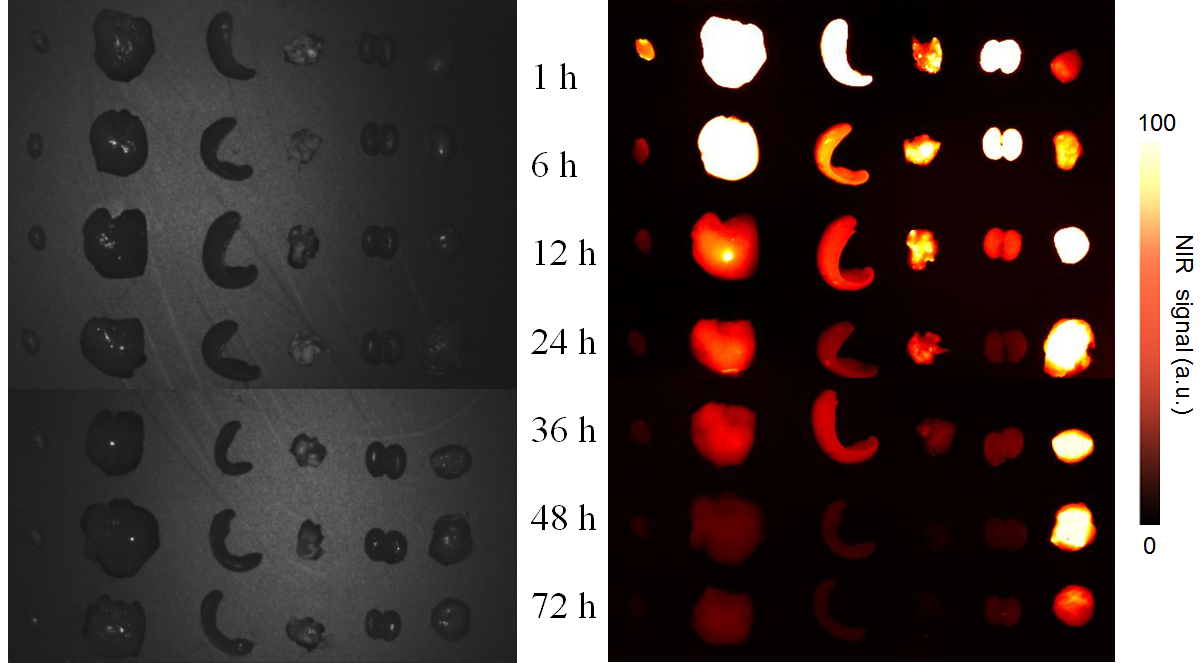


**Figure S3.** Bright-field (left) and NIR-II fluorescent images (right) of main organs and tumors after tail vein injection of oMSN-GNR-ICG for different durations.


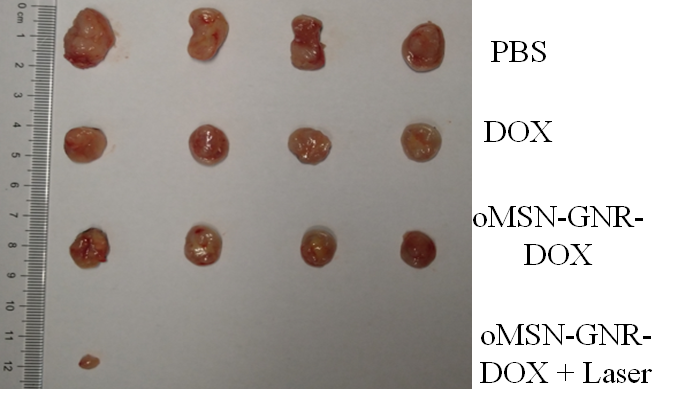


**Figure S4.** Digital photos of huh7 liver tumors after 15 days of different treatments of PBS, DOX, oMSN-GNR-DOX, oMSN-GNR-DOX + laser.


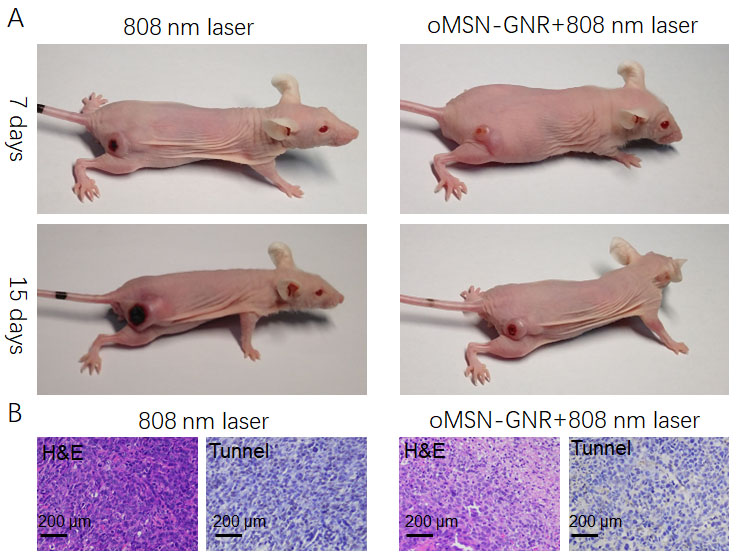


**Figure S5.** Typical digital images of huh7 tumor-bearing nude mice after treatment of 808 nm laser or oMSN-GNR +laser for 15 days (A). H&E and Tunnel staining of 808 nm laser or oMSN-GNR +laser treatment for 5 days (B).


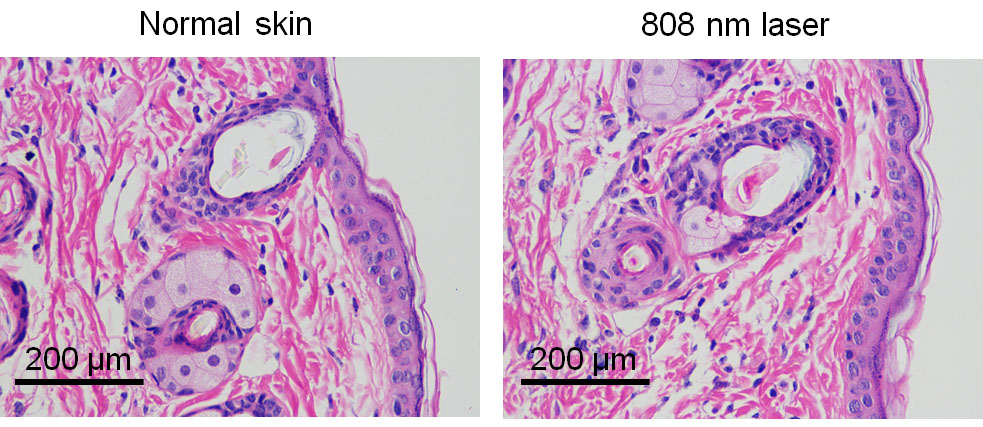


**Figure S6.** H&E staining images of normal skin and skin after 808 nm laser irradiation.


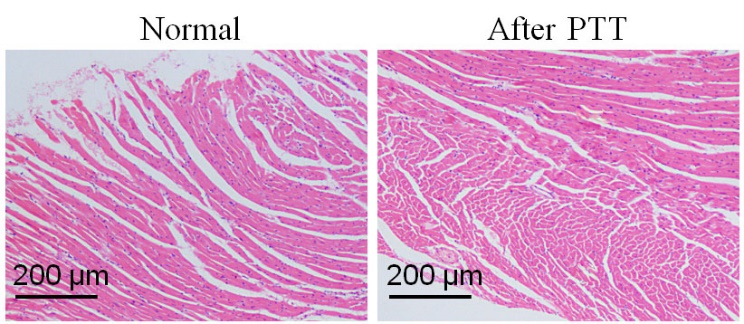


**Figure S7.** H & E staining of normal muscle tissue and tumor adjacent muscle tissue after PTT.


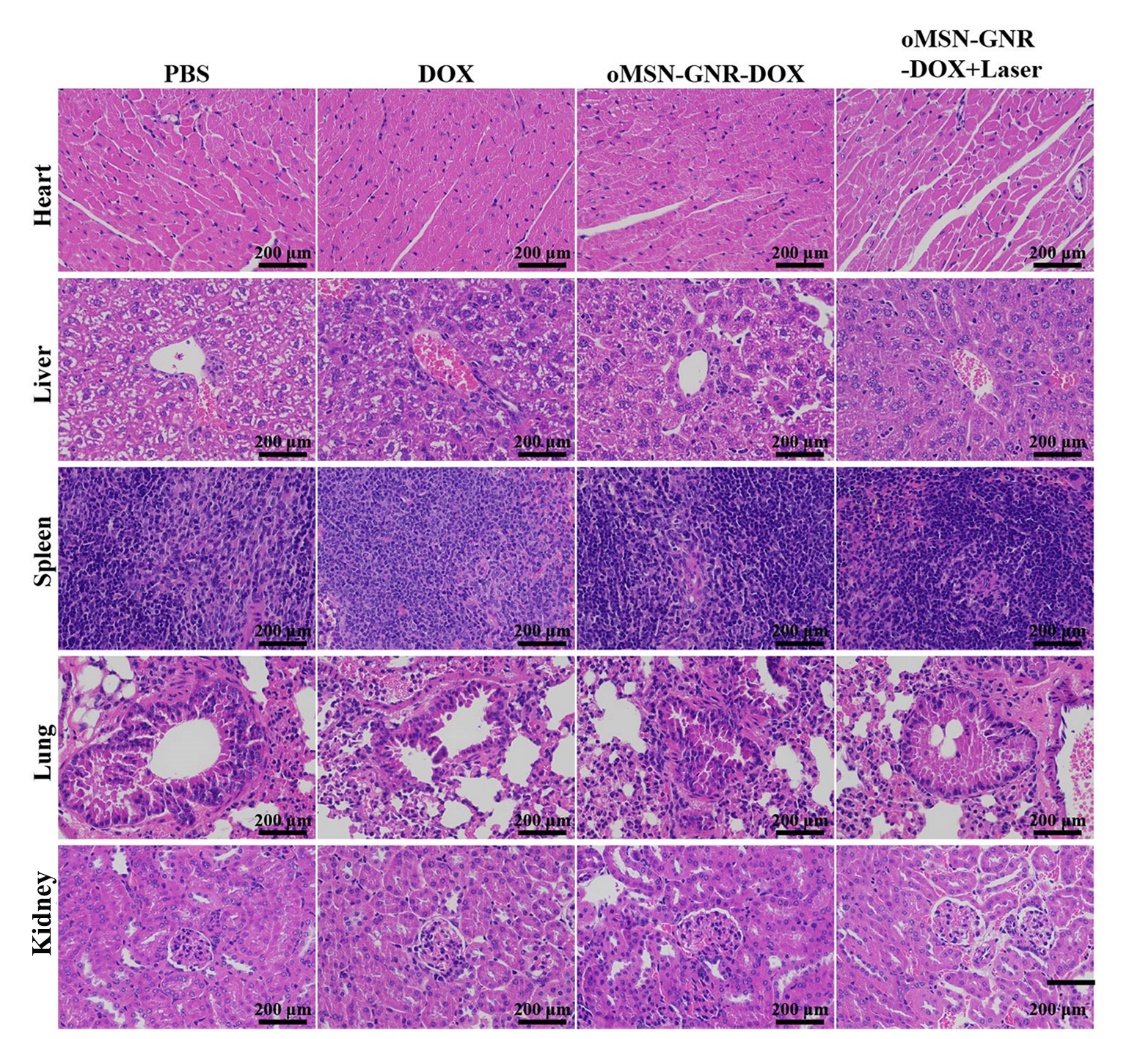


**Figure S8.** H & E staining images of main organs after excised at 15 days various treatments of PBS, DOX, oMSN-GNR-DOX, oMSN-GNR-DOX + laser.


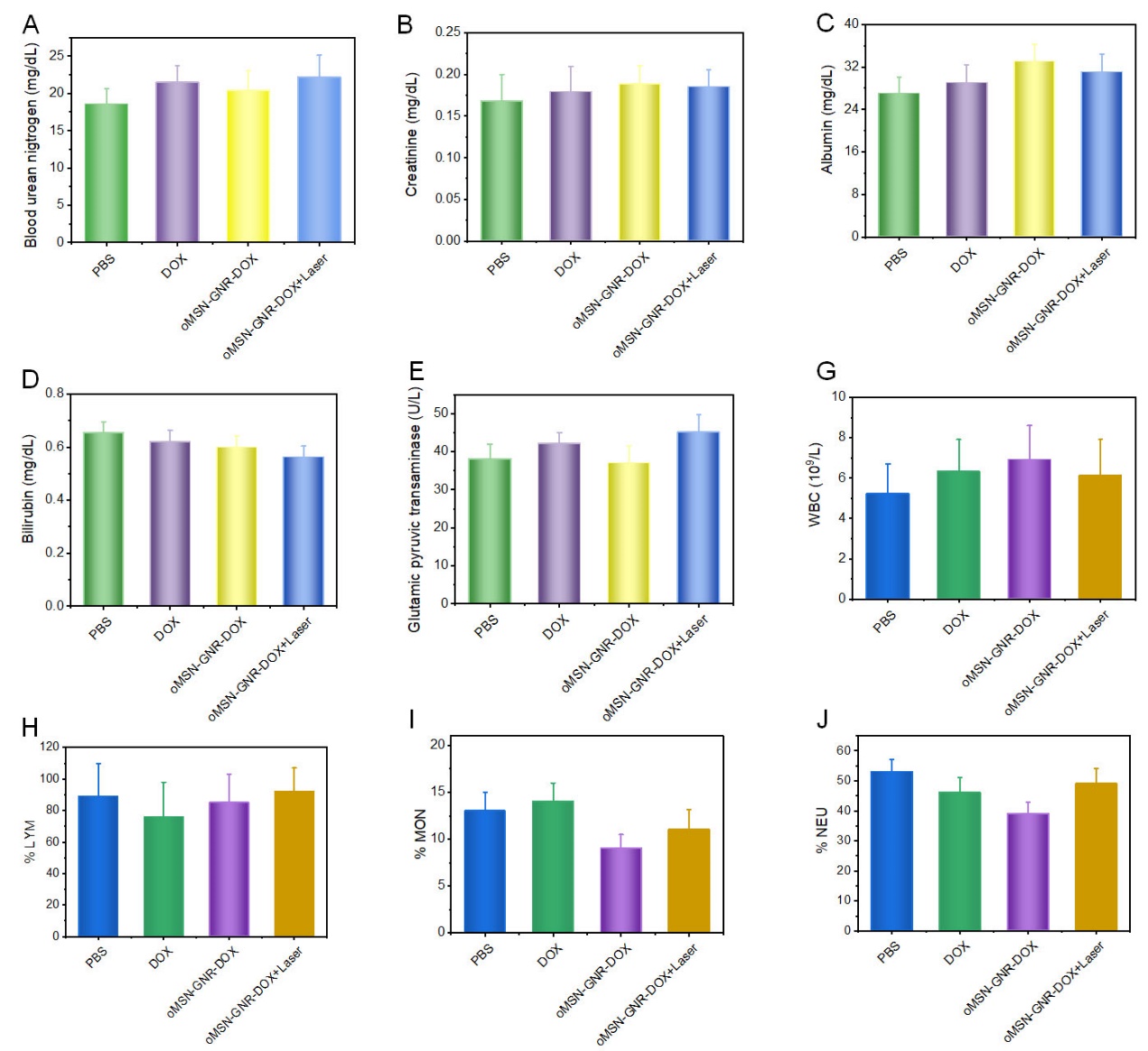


**Figure S9.** All corresponding blood biochemistry (A-F: blood urea nitrogen, Creatinine, Albumin, Bilirubin, Glutamic pyruvic transaminase) and blood routine factors (H-J: white blood cells, lymphocytes, monocytes, neutrophils) of PBS, DOX, oMSN-GNR-DOX and oMSN-GNR-DOX + laser for 15 days.
